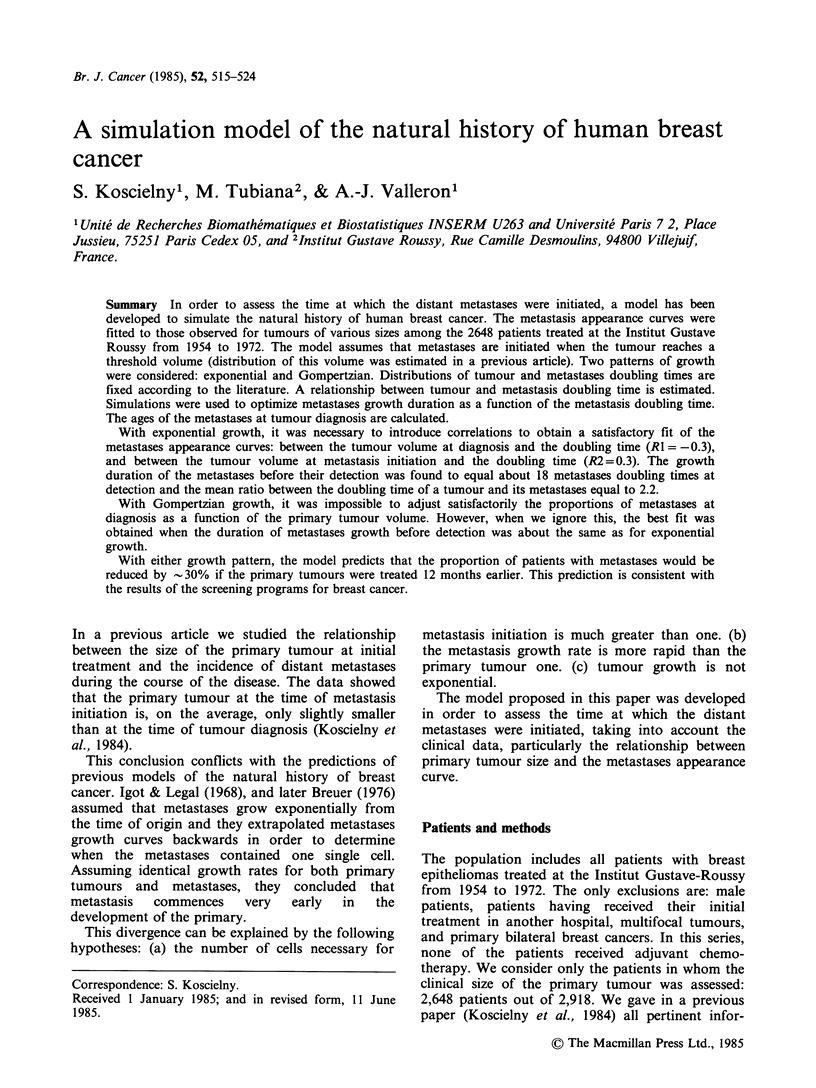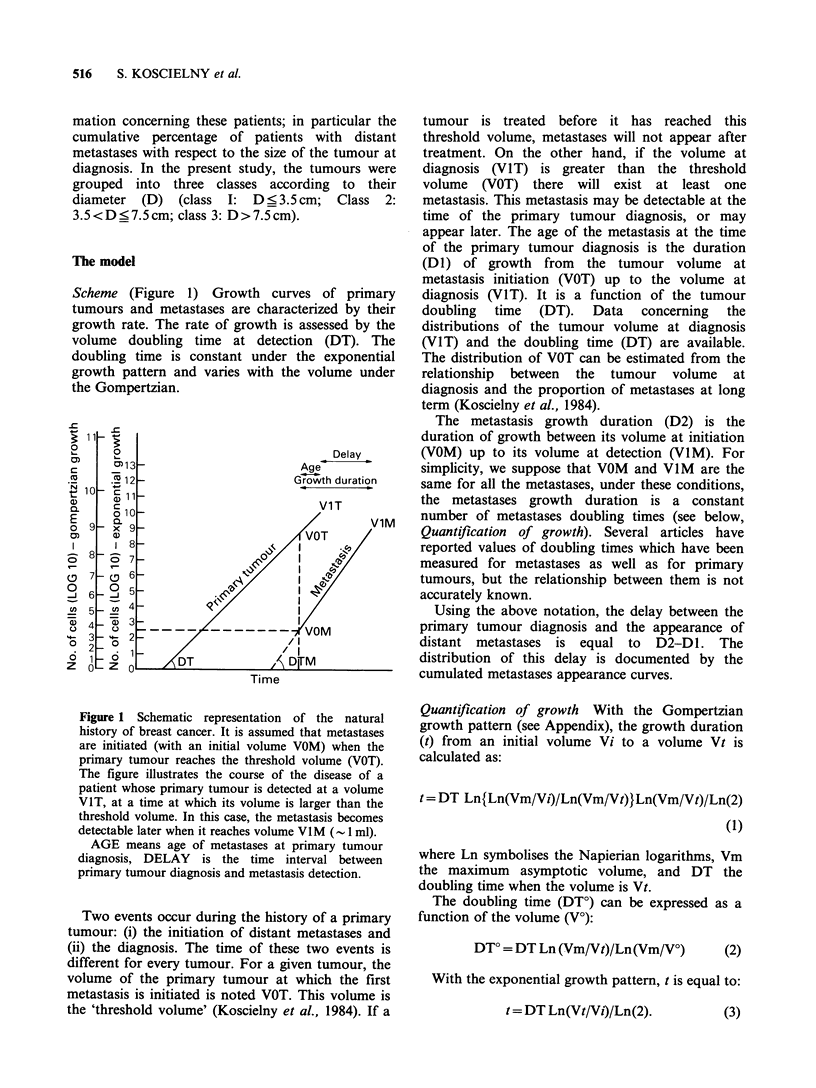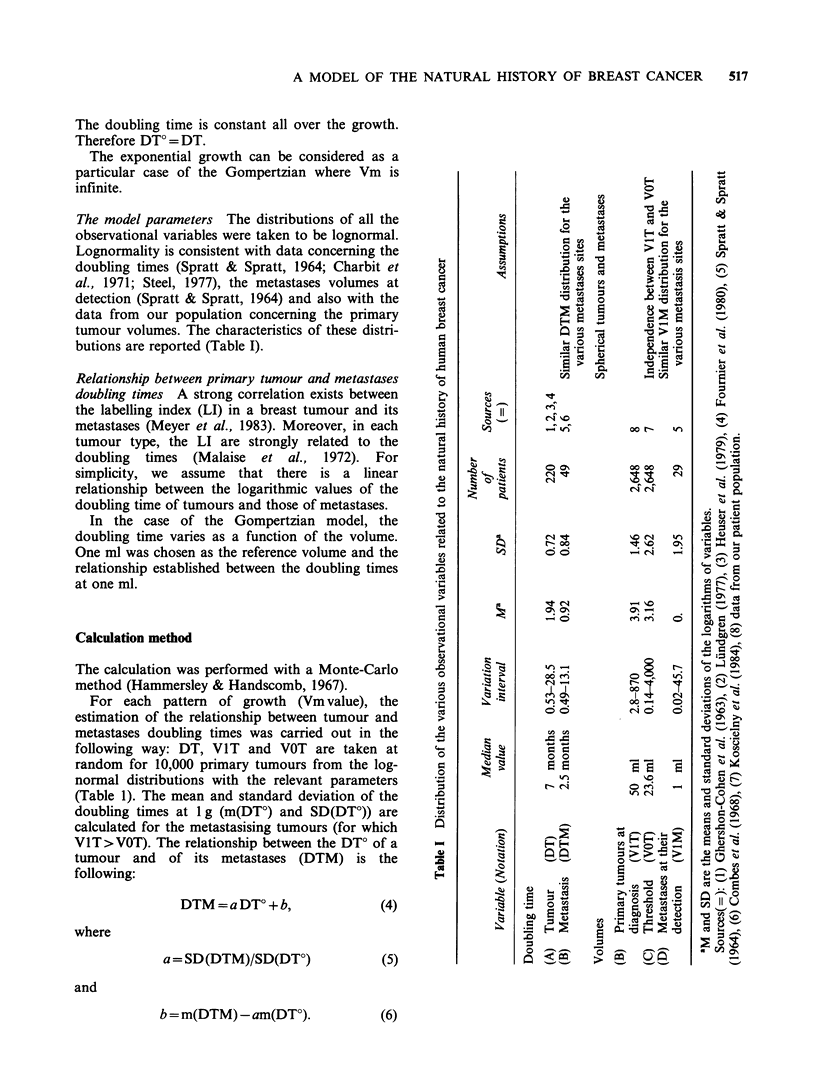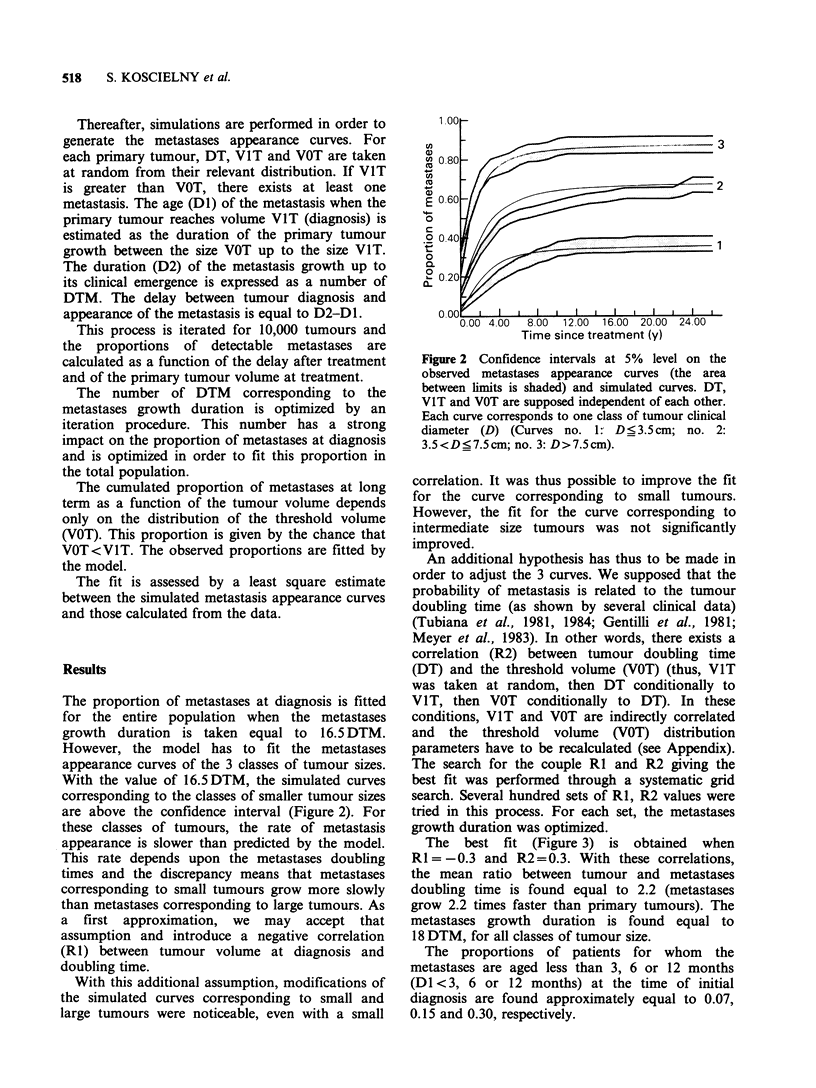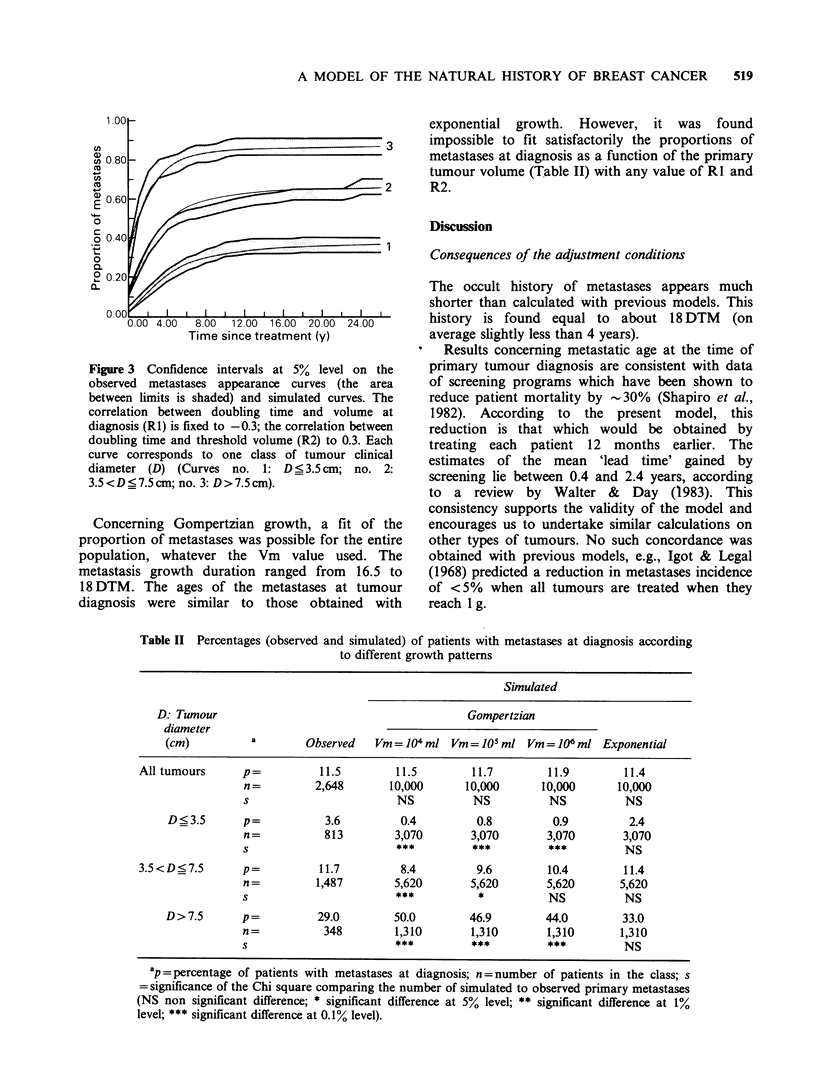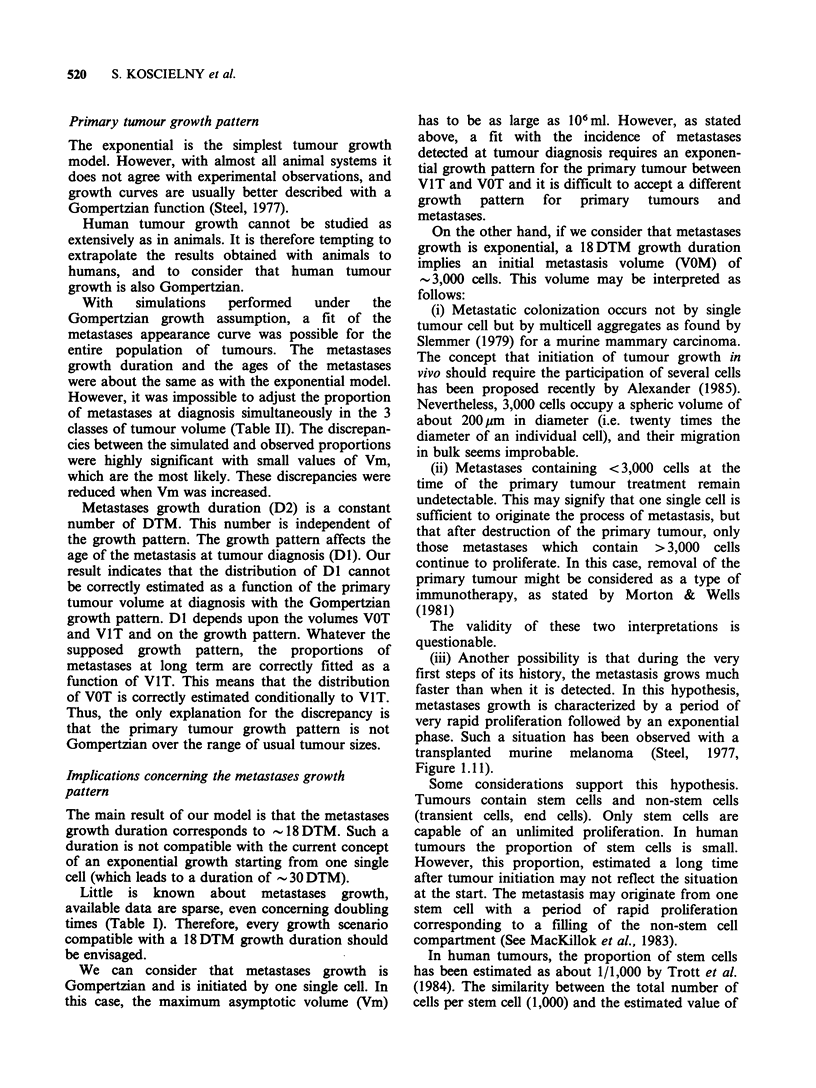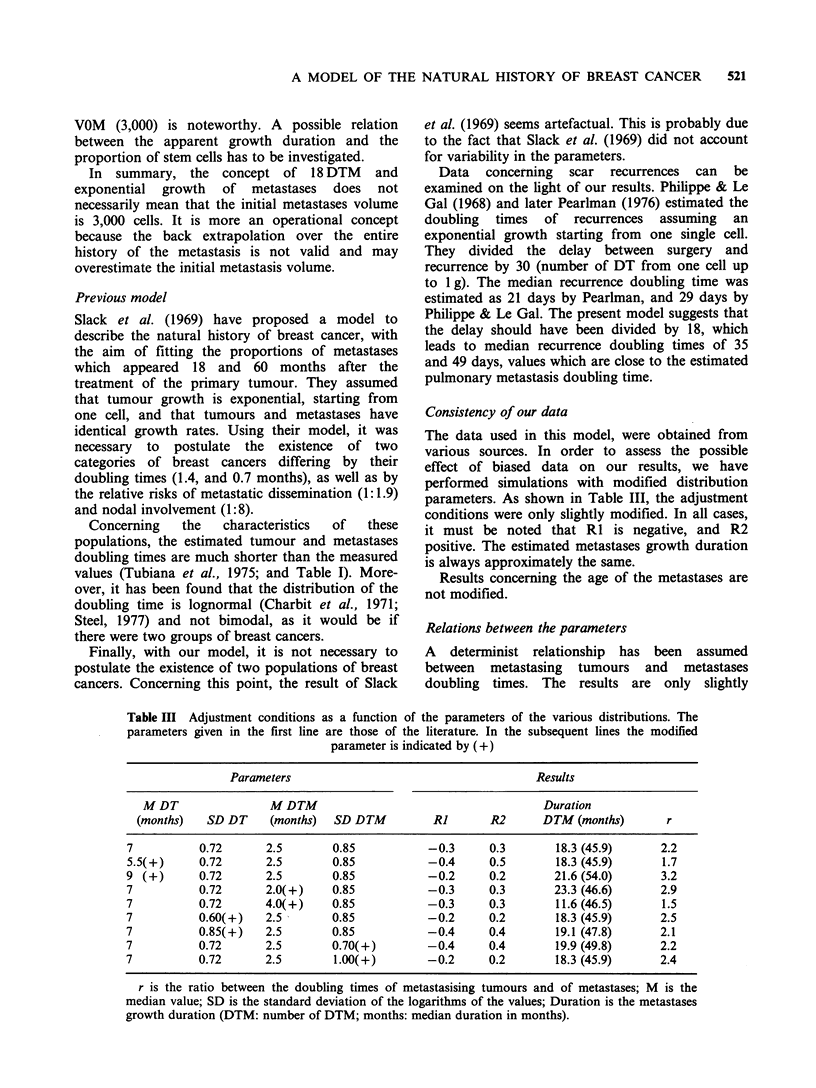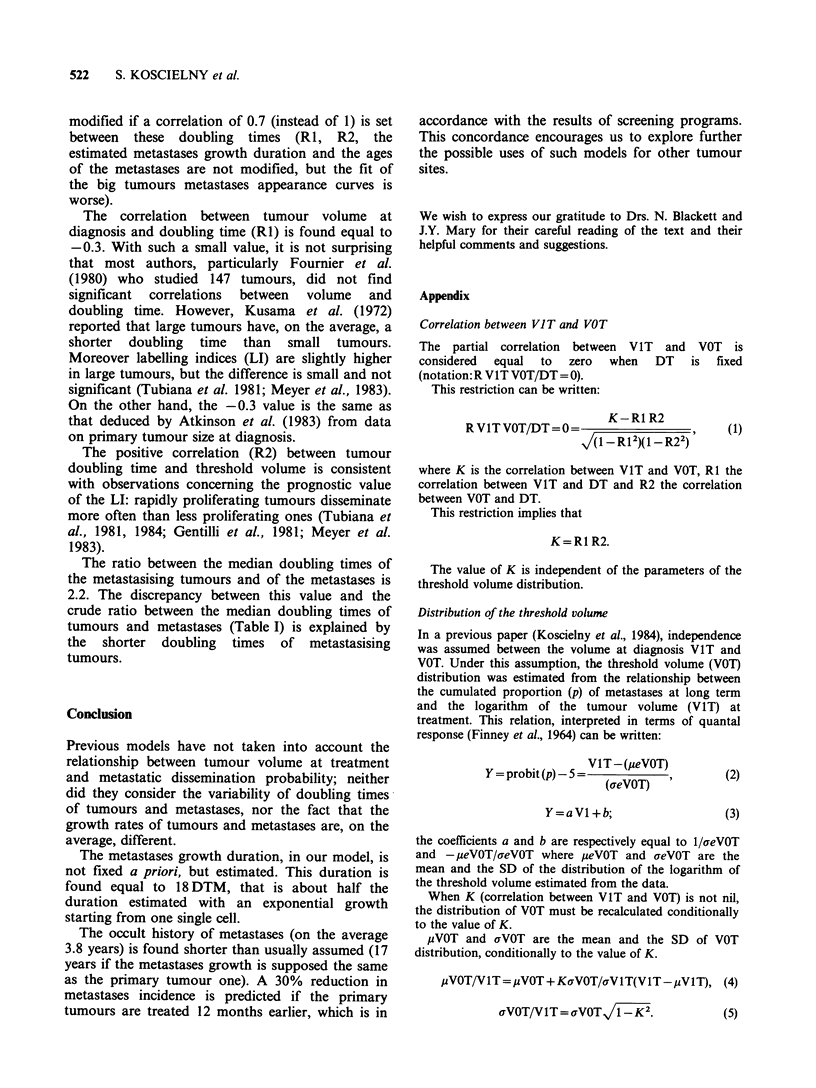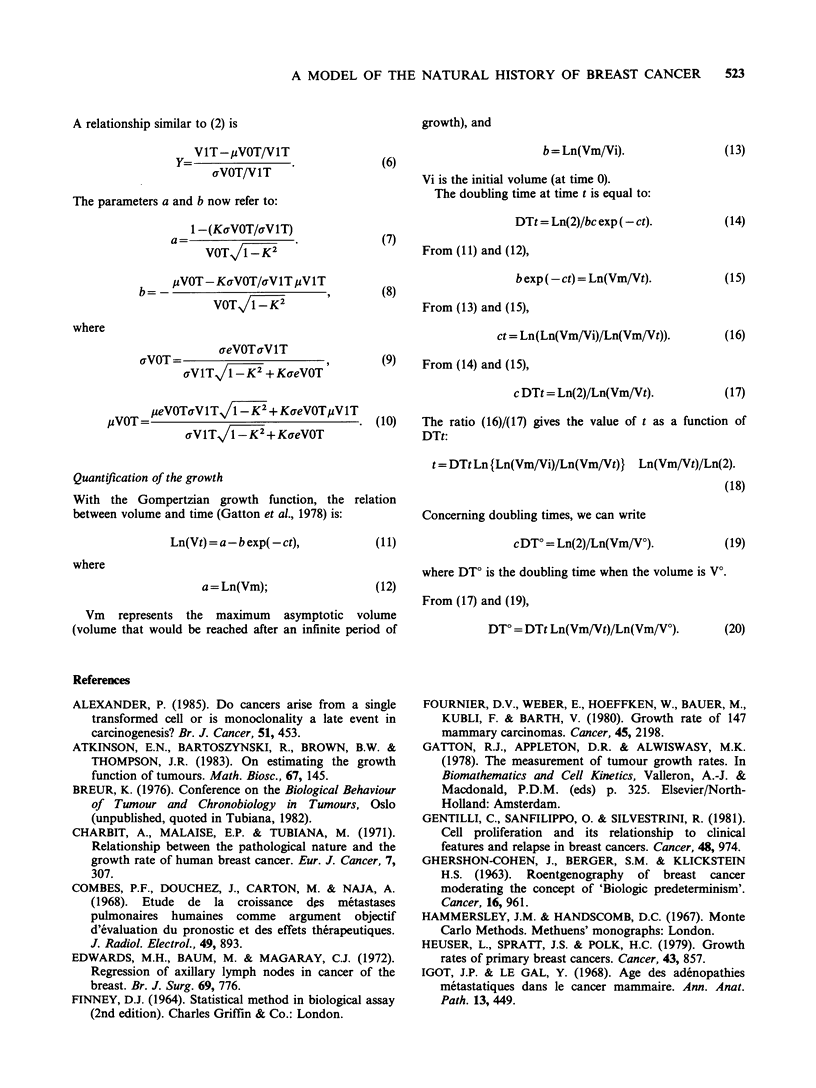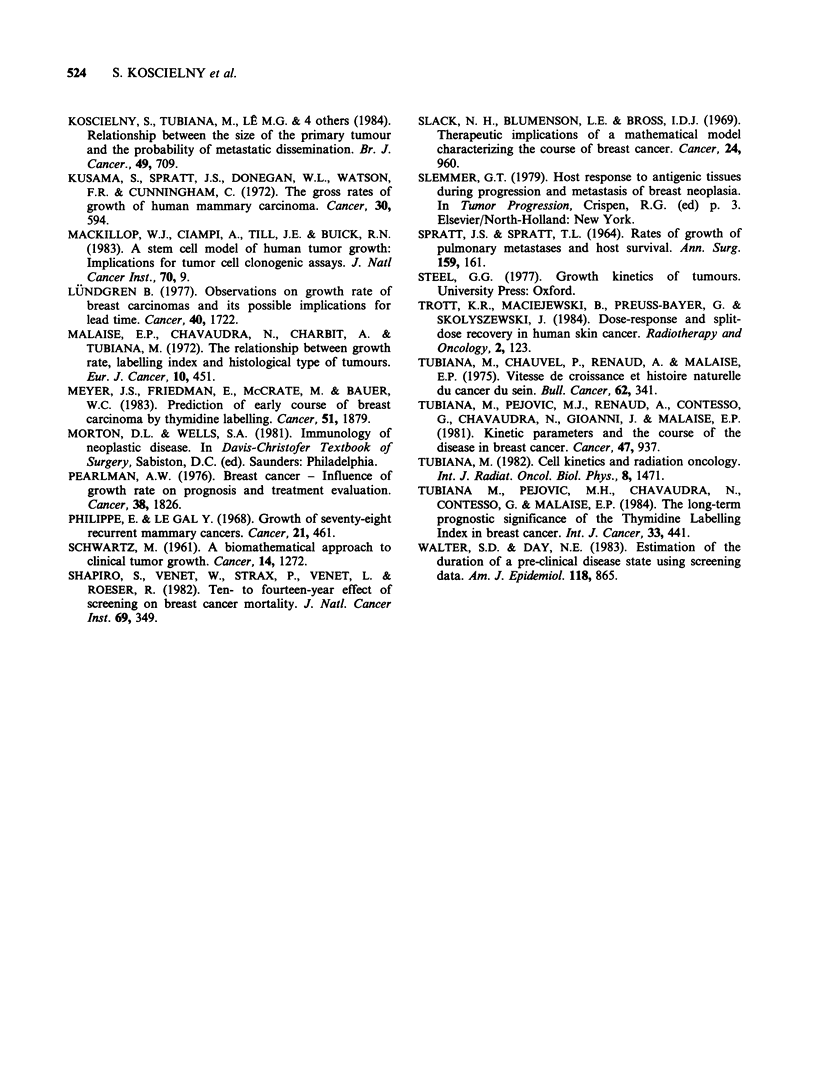# A simulation model of the natural history of human breast cancer.

**DOI:** 10.1038/bjc.1985.222

**Published:** 1985-10

**Authors:** S. Koscielny, M. Tubiana, A. J. Valleron

## Abstract

In order to assess the time at which the distant metastases were initiated, a model has been developed to simulate the natural history of human breast cancer. The metastasis appearance curves were fitted to those observed for tumours of various sizes among the 2648 patients treated at the Institut Gustave Roussy from 1954 to 1972. The model assumes that metastases are initiated when the tumour reaches a threshold volume (distribution of this volume was estimated in a previous article). Two patterns of growth were considered: exponential and Gompertzian. Distributions of tumour and metastases doubling times are fixed according to the literature. A relationship between tumour and metastasis doubling time is estimated. Simulations were used to optimize metastases growth duration as a function of the metastasis doubling time. The ages of the metastases at tumour diagnosis are calculated. With exponential growth, it was necessary to introduce correlations to obtain a satisfactory fit of the metastases appearance curves: between the tumour volume at diagnosis and the doubling time (R1 = -0.3), and between the tumour volume at metastasis initiation and the doubling time (R2 = 0.3). The growth duration of the metastases before their detection was found to equal about 18 metastases doubling times at detection and the mean ratio between the doubling time of a tumour and its metastases equal to 2.2. With Gompertzian growth, it was impossible to adjust satisfactorily the proportions of metastases at diagnosis as a function of the primary tumour volume. However, when we ignore this, the best fit was obtained when the duration of metastases growth before detection was about the same as for exponential growth. With either growth pattern, the model predicts that the proportion of patients with metastases would be reduced by approximately 30% if the primary tumours were treated 12 months earlier. This prediction is consistent with the results of the screening programs for breast cancer.


*Br. J. Cancer* (1985), **52**, 515–524

### A simulation model of the natural history of human breast cancer

S. Koscielny[1], M. Tubiana[2], & A.-J. Valleron[1]

[1]*Unité de Recherches Biomathématiques et Biostatistiques INSERM U263 and Université Paris 7 2, Place Jussieu, 75251 Paris Cedex 05, and* [2]*Institut Gustave Roussy, Rue Camille Desmoulins, 94800 Villejuif, France.*

**Summary** In order to assess the time at which the distant metastases were initiated, a model has been developed to simulate the natural history of human breast cancer. The metastasis appearance curves were fitted to those observed for tumours of various sizes among the 2648 patients treated at the Institut Gustave Roussy from 1954 to 1972. The model assumes that metastases are initiated when the tumour reaches a threshold volume (distribution of this volume was estimated in a previous article). Two patterns of growth were considered: exponential and Gompertzian. Distributions of tumour and metastases doubling times are fixed according to the literature. A relationship between tumour and metastasis doubling time is estimated. Simulations were used to optimize metastases growth duration as a function of the metastasis doubling time. The ages of the metastases at tumour diagnosis are calculated.

With exponential growth, it was necessary to introduce correlations to obtain a satisfactory fit of the metastases appearance curves: between the tumour volume at diagnosis and the doubling time ($R1 = -0.3$), and between the tumour volume at metastasis initiation and the doubling time ($R2 = 0.3$). The growth duration of the metastases before their detection was found to equal about 18 metastases doubling times at detection and the mean ratio between the doubling time of a tumour and its metastases equal to 2.2.

With Gompertzian growth, it was impossible to adjust satisfactorily the proportions of metastases at diagnosis as a function of the primary tumour volume. However, when we ignore this, the best fit was obtained when the duration of metastases growth before detection was about the same as for exponential growth.

With either growth pattern, the model predicts that the proportion of patients with metastases would be reduced by $\sim$30% if the primary tumours were treated 12 months earlier. This prediction is consistent with the results of the screening programs for breast cancer.

In a previous article we studied the relationship between the size of the primary tumour at initial treatment and the incidence of distant metastases during the course of the disease. The data showed that the primary tumour at the time of metastasis initiation is, on the average, only slightly smaller than at the time of tumour diagnosis (Koscielny *et al.,* 1984).

This conclusion conflicts with the predictions of previous models of the natural history of breast cancer. Igot & Legal (1968), and later Breuer (1976) assumed that metastases grow exponentially from the time of origin and they extrapolated metastases growth curves backwards in order to determine when the metastases contained one single cell. Assuming identical growth rates for both primary tumours and metastases, they concluded that metastasis commences very early in the development of the primary.

This divergence can be explained by the following hypotheses: (a) the number of cells necessary for metastasis initiation is much greater than one. (b) the metastasis growth rate is more rapid than the primary tumour one. (c) tumour growth is not exponential.

The model proposed in this paper was developed in order to assess the time at which the distant metastases were initiated, taking into account the clinical data, particularly the relationship between primary tumour size and the metastases appearance curve.

### Patients and methods

The population includes all patients with breast epitheliomas treated at the Institut Gustave-Roussy from 1954 to 1972. The only exclusions are: male patients, patients having received their initial treatment in another hospital, multifocal tumours, and primary bilateral breast cancers. In this series, none of the patients received adjuvant chemotherapy. We consider only the patients in whom the clinical size of the primary tumour was assessed: 2,648 patients out of 2,918. We gave in a previous paper (Koscielny *et al.,* 1984) all pertinent infor-

Correspondence: S. Koscielny.
Received 1 January 1985; and in revised form, 11 June 1985.

© The Macmillan Press Ltd., 1985

mation concerning these patients; in particular the cumulative percentage of patients with distant metastases with respect to the size of the tumour at diagnosis. In the present study, the tumours were grouped into three classes according to their diameter (D) (class I: $D \leq 3.5$ cm; Class 2: $3.5 < D \leq 7.5$ cm; class 3: $D > 7.5$ cm).

### The model

*Scheme* (Figure 1) Growth curves of primary tumours and metastases are characterized by their growth rate. The rate of growth is assessed by the volume doubling time at detection (DT). The doubling time is constant under the exponential growth pattern and varies with the volume under the Gompertzian.

**Figure 1** Schematic representation of the natural history of breast cancer. It is assumed that metastases are initiated (with an initial volume V0M) when the primary tumour reaches the threshold volume (V0T). The figure illustrates the course of the disease of a patient whose primary tumour is detected at a volume V1T, at a time at which its volume is larger than the threshold volume. In this case, the metastasis becomes detectable later when it reaches volume V1M ($\sim$1 ml).

AGE means age of metastases at primary tumour diagnosis, DELAY is the time interval between primary tumour diagnosis and metastasis detection.

Two events occur during the history of a primary tumour: (i) the initiation of distant metastases and (ii) the diagnosis. The time of these two events is different for every tumour. For a given tumour, the volume of the primary tumour at which the first metastasis is initiated is noted V0T. This volume is the 'threshold volume' (Koscielny *et al.*, 1984). If a tumour is treated before it has reached this threshold volume, metastases will not appear after treatment. On the other hand, if the volume at diagnosis (V1T) is greater than the threshold volume (V0T) there will exist at least one metastasis. This metastasis may be detectable at the time of the primary tumour diagnosis, or may appear later. The age of the metastasis at the time of the primary tumour diagnosis is the duration (D1) of growth from the tumour volume at metastasis initiation (V0T) up to the volume at diagnosis (V1T). It is a function of the tumour doubling time (DT). Data concerning the distributions of the tumour volume at diagnosis (V1T) and the doubling time (DT) are available. The distribution of V0T can be estimated from the relationship between the tumour volume at diagnosis and the proportion of metastases at long term (Koscielny *et al.*, 1984).

The metastasis growth duration (D2) is the duration of growth between its volume at initiation (V0M) up to its volume at detection (V1M). For simplicity, we suppose that V0M and V1M are the same for all the metastases, under these conditions, the metastases growth duration is a constant number of metastases doubling times (see below, *Quantification of growth*). Several articles have reported values of doubling times which have been measured for metastases as well as for primary tumours, but the relationship between them is not accurately known.

Using the above notation, the delay between the primary tumour diagnosis and the appearance of distant metastases is equal to D2–D1. The distribution of this delay is documented by the cumulated metastases appearance curves.

*Quantification of growth* With the Gompertzian growth pattern (see Appendix), the growth duration ($t$) from an initial volume V$i$ to a volume V$t$ is calculated as:

$$t = \text{DT Ln}\{\text{Ln}(\text{Vm}/\text{V}i)/\text{Ln}(\text{Vm}/\text{V}t)\}\text{Ln}(\text{Vm}/\text{V}t)/\text{Ln}(2) \quad (1)$$

where Ln symbolises the Napierian logarithms, Vm the maximum asymptotic volume, and DT the doubling time when the volume is V$t$.

The doubling time (DT°) can be expressed as a function of the volume (V°):

$$\text{DT}° = \text{DT Ln}(\text{Vm}/\text{V}t)/\text{Ln}(\text{Vm}/\text{V}°) \quad (2)$$

With the exponential growth pattern, $t$ is equal to:

$$t = \text{DT Ln}(\text{V}t/\text{V}i)/\text{Ln}(2). \quad (3)$$

The doubling time is constant all over the growth. Therefore DT° = DT.

The exponential growth can be considered as a particular case of the Gompertzian where Vm is infinite.

*The model parameters* The distributions of all the observational variables were taken to be lognormal. Lognormality is consistent with data concerning the doubling times (Spratt & Spratt, 1964; Charbit *et al.*, 1971; Steel, 1977), the metastases volumes at detection (Spratt & Spratt, 1964) and also with the data from our population concerning the primary tumour volumes. The characteristics of these distributions are reported (Table I).

*Relationship between primary tumour and metastases doubling times* A strong correlation exists between the labelling index (LI) in a breast tumour and its metastases (Meyer *et al.*, 1983). Moreover, in each tumour type, the LI are strongly related to the doubling times (Malaise *et al.*, 1972). For simplicity, we assume that there is a linear relationship between the logarithmic values of the doubling time of tumours and those of metastases.

In the case of the Gompertzian model, the doubling time varies as a function of the volume. One ml was chosen as the reference volume and the relationship established between the doubling times at one ml.

### Calculation method

The calculation was performed with a Monte-Carlo method (Hammersley & Handscomb, 1967).

For each pattern of growth (Vm value), the estimation of the relationship between tumour and metastases doubling times was carried out in the following way: DT, V1T and V0T are taken at random for 10,000 primary tumours from the lognormal distributions with the relevant parameters (Table 1). The mean and standard deviation of the doubling times at 1 g (m(DT°) and SD(DT°)) are calculated for the metastasising tumours (for which V1T > V0T). The relationship between the DT° of a tumour and of its metastases (DTM) is the following:

$$DTM = a\,DT^{\circ} + b, \qquad (4)$$

where

$$a = SD(DTM)/SD(DT^{\circ}) \qquad (5)$$

and

$$b = m(DTM) - a\,m(DT^{\circ}). \qquad (6)$$

**Table I** Distribution of the various observational variables related to the natural history of human breast cancer

| *Variable (Notation)* | *Median value* | *Variation interval* | *M*[a] | *SD*[a] | *Number of patients* | *Sources (=)* | *Assumptions* |
|---|---|---|---|---|---|---|---|
| Doubling time | | | | | | | |
| (A) Tumour (DT) | 7 months | 0.53–28.5 | 1.94 | 0.72 | 220 | 1,2,3,4 | Similar DTM distribution for the various metastases sites |
| (B) Metastasis (DTM) | 2.5 months | 0.49–13.1 | 0.92 | 0.84 | 49 | 5,6 | |
| Volumes | | | | | | | Spherical tumours and metastases |
| (B) Primary tumours at diagnosis (V1T) | 50 ml | 2.8–870 | 3.91 | 1.46 | 2,648 | 8 | |
| (C) Threshold (V0T) | 23.6 ml | 0.14–4,000 | 3.16 | 2.62 | 2,648 | 7 | Independence between V1T and V0T |
| (D) Metastases at their detection (V1M) | 1 ml | 0.02–45.7 | 0. | 1.95 | 29 | 5 | Similar V1M distribution for the various metastasis sites |

[a]M and SD are the means and standard deviations of the logarithms of variables.

Sources(=): (1) Ghershon-Cohen *et al.* (1963), (2) Lündgren (1977), (3) Heuser *et al.* (1979), (4) Fournier *et al.* (1980), (5) Spratt & Spratt (1964), (6) Combes *et al.* (1968), (7) Koscielny *et al.* (1984), (8) data from our patient population.

Thereafter, simulations are performed in order to generate the metastases appearance curves. For each primary tumour, DT, V1T and V0T are taken at random from their relevant distribution. If V1T is greater than V0T, there exists at least one metastasis. The age (D1) of the metastasis when the primary tumour reaches volume V1T (diagnosis) is estimated as the duration of the primary tumour growth between the size V0T up to the size V1T. The duration (D2) of the metastasis growth up to its clinical emergence is expressed as a number of DTM. The delay between tumour diagnosis and appearance of the metastasis is equal to D2–D1.

This process is iterated for 10,000 tumours and the proportions of detectable metastases are calculated as a function of the delay after treatment and of the primary tumour volume at treatment.

The number of DTM corresponding to the metastases growth duration is optimized by an iteration procedure. This number has a strong impact on the proportion of metastases at diagnosis and is optimized in order to fit this proportion in the total population.

The cumulated proportion of metastases at long term as a function of the tumour volume depends only on the distribution of the threshold volume (V0T). This proportion is given by the chance that V0T<V1T. The observed proportions are fitted by the model.

The fit is assessed by a least square estimate between the simulated metastasis appearance curves and those calculated from the data.

### Results

The proportion of metastases at diagnosis is fitted for the entire population when the metastases growth duration is taken equal to 16.5 DTM. However, the model has to fit the metastases appearance curves of the 3 classes of tumour sizes. With the value of 16.5 DTM, the simulated curves corresponding to the classes of smaller tumour sizes are above the confidence interval (Figure 2). For these classes of tumours, the rate of metastasis appearance is slower than predicted by the model. This rate depends upon the metastases doubling times and the discrepancy means that metastases corresponding to small tumours grow more slowly than metastases corresponding to large tumours. As a first approximation, we may accept that assumption and introduce a negative correlation (R1) between tumour volume at diagnosis and doubling time.

With this additional assumption, modifications of the simulated curves corresponding to small and large tumours were noticeable, even with a small

**Figure 2** Confidence intervals at 5% level on the observed metastases appearance curves (the area between limits is shaded) and simulated curves. DT, V1T and V0T are supposed independent of each other. Each curve corresponds to one class of tumour clinical diameter (*D*) (Curves no. 1: $D \leqq 3.5$ cm; no. 2: $3.5 < D \leqq 7.5$ cm; no. 3: $D > 7.5$ cm).

correlation. It was thus possible to improve the fit for the curve corresponding to small tumours. However, the fit for the curve corresponding to intermediate size tumours was not significantly improved.

An additional hypothesis has thus to be made in order to adjust the 3 curves. We supposed that the probability of metastasis is related to the tumour doubling time (as shown by several clinical data) (Tubiana *et al.*, 1981, 1984; Gentilli *et al.*, 1981; Meyer *et al.*, 1983). In other words, there exists a correlation (R2) between tumour doubling time (DT) and the threshold volume (V0T) (thus, V1T was taken at random, then DT conditionally to V1T, then V0T conditionally to DT). In these conditions, V1T and V0T are indirectly correlated and the threshold volume (V0T) distribution parameters have to be recalculated (see Appendix). The search for the couple R1 and R2 giving the best fit was performed through a systematic grid search. Several hundred sets of R1, R2 values were tried in this process. For each set, the metastases growth duration was optimized.

The best fit (Figure 3) is obtained when $R1 = -0.3$ and $R2 = 0.3$. With these correlations, the mean ratio between tumour and metastases doubling time is found equal to 2.2 (metastases grow 2.2 times faster than primary tumours). The metastases growth duration is found equal to 18 DTM, for all classes of tumour size.

The proportions of patients for whom the metastases are aged less than 3, 6 or 12 months (D1<3, 6 or 12 months) at the time of initial diagnosis are found approximately equal to 0.07, 0.15 and 0.30, respectively.

**Figure 3** Confidence intervals at 5% level on the observed metastases appearance curves (the area between limits is shaded) and simulated curves. The correlation between doubling time and volume at diagnosis (R1) is fixed to −0.3; the correlation between doubling time and threshold volume (R2) to 0.3. Each curve corresponds to one class of tumour clinical diameter (*D*) (Curves no. 1: $D \leqq 3.5$ cm; no. 2: $3.5 < D \leqq 7.5$ cm; no. 3: $D > 7.5$ cm).

Concerning Gompertzian growth, a fit of the proportion of metastases was possible for the entire population, whatever the Vm value used. The metastasis growth duration ranged from 16.5 to 18 DTM. The ages of the metastases at tumour diagnosis were similar to those obtained with exponential growth. However, it was found impossible to fit satisfactorily the proportions of metastases at diagnosis as a function of the primary tumour volume (Table II) with any value of R1 and R2.

### Discussion

### *Consequences of the adjustment conditions*

The occult history of metastases appears much shorter than calculated with previous models. This history is found equal to about 18 DTM (on average slightly less than 4 years).

Results concerning metastatic age at the time of primary tumour diagnosis are consistent with data of screening programs which have been shown to reduce patient mortality by ~30% (Shapiro *et al.*, 1982). According to the present model, this reduction is that which would be obtained by treating each patient 12 months earlier. The estimates of the mean 'lead time' gained by screening lie between 0.4 and 2.4 years, according to a review by Walter & Day (1983). This consistency supports the validity of the model and encourages us to undertake similar calculations on other types of tumours. No such concordance was obtained with previous models, e.g., Igot & Legal (1968) predicted a reduction in metastases incidence of <5% when all tumours are treated when they reach 1 g.

**Table II** Percentages (observed and simulated) of patients with metastases at diagnosis according to different growth patterns

| | | | *Simulated* | | | |
|---|---|---|---|---|---|---|
| | | | *Gompertzian* | | | |
| *D: Tumour diameter (cm)* | [a] | *Observed* | *Vm = $10^4$ ml* | *Vm = $10^5$ ml* | *Vm = $10^6$ ml* | *Exponential* |
| All tumours | *p* = | 11.5 | 11.5 | 11.7 | 11.9 | 11.4 |
| | *n* = | 2,648 | 10,000 | 10,000 | 10,000 | 10,000 |
| | *s* | | NS | NS | NS | NS |
| $D \leqq 3.5$ | *p* = | 3.6 | 0.4 | 0.8 | 0.9 | 2.4 |
| | *n* = | 813 | 3,070 | 3,070 | 3,070 | 3,070 |
| | *s* | | *** | *** | *** | NS |
| $3.5 < D \leqq 7.5$ | *p* = | 11.7 | 8.4 | 9.6 | 10.4 | 11.4 |
| | *n* = | 1,487 | 5,620 | 5,620 | 5,620 | 5,620 |
| | *s* | | *** | * | NS | NS |
| $D > 7.5$ | *p* = | 29.0 | 50.0 | 46.9 | 44.0 | 33.0 |
| | *n* = | 348 | 1,310 | 1,310 | 1,310 | 1,310 |
| | *s* | | *** | *** | *** | NS |

[a]*p* = percentage of patients with metastases at diagnosis; *n* = number of patients in the class; *s* = significance of the Chi square comparing the number of simulated to observed primary metastases (NS non significant difference; * significant difference at 5% level; ** significant difference at 1% level; *** significant difference at 0.1% level).

*Primary tumour growth pattern*

The exponential is the simplest tumour growth model. However, with almost all animal systems it does not agree with experimental observations, and growth curves are usually better described with a Gompertzian function (Steel, 1977).

Human tumour growth cannot be studied as extensively as in animals. It is therefore tempting to extrapolate the results obtained with animals to humans, and to consider that human tumour growth is also Gompertzian.

With simulations performed under the Gompertzian growth assumption, a fit of the metastases appearance curve was possible for the entire population of tumours. The metastases growth duration and the ages of the metastases were about the same as with the exponential model. However, it was impossible to adjust the proportion of metastases at diagnosis simultaneously in the 3 classes of tumour volume (Table II). The discrepancies between the simulated and observed proportions were highly significant with small values of Vm, which are the most likely. These discrepancies were reduced when Vm was increased.

Metastases growth duration (D2) is a constant number of DTM. This number is independent of the growth pattern. The growth pattern affects the age of the metastasis at tumour diagnosis (D1). Our result indicates that the distribution of D1 cannot be correctly estimated as a function of the primary tumour volume at diagnosis with the Gompertzian growth pattern. D1 depends upon the volumes V0T and V1T and on the growth pattern. Whatever the supposed growth pattern, the proportions of metastases at long term are correctly fitted as a function of V1T. This means that the distribution of V0T is correctly estimated conditionally to V1T. Thus, the only explanation for the discrepancy is that the primary tumour growth pattern is not Gompertzian over the range of usual tumour sizes.

*Implications concerning the metastases growth pattern*

The main result of our model is that the metastases growth duration corresponds to ~18 DTM. Such a duration is not compatible with the current concept of an exponential growth starting from one single cell (which leads to a duration of ~30 DTM).

Little is known about metastases growth, available data are sparse, even concerning doubling times (Table I). Therefore, every growth scenario compatible with a 18 DTM growth duration should be envisaged.

We can consider that metastases growth is Gompertzian and is initiated by one single cell. In this case, the maximum asymptotic volume (Vm) has to be as large as $10^6$ ml. However, as stated above, a fit with the incidence of metastases detected at tumour diagnosis requires an exponential growth pattern for the primary tumour between V1T and V0T and it is difficult to accept a different growth pattern for primary tumours and metastases.

On the other hand, if we consider that metastases growth is exponential, a 18 DTM growth duration implies an initial metastasis volume (V0M) of ~3,000 cells. This volume may be interpreted as follows:

(i) Metastatic colonization occurs not by single tumour cell but by multicell aggregates as found by Slemmer (1979) for a murine mammary carcinoma. The concept that initiation of tumour growth *in vivo* should require the participation of several cells has been proposed recently by Alexander (1985). Nevertheless, 3,000 cells occupy a spheric volume of about 200 μm in diameter (i.e. twenty times the diameter of an individual cell), and their migration in bulk seems improbable.

(ii) Metastases containing <3,000 cells at the time of the primary tumour treatment remain undetectable. This may signify that one single cell is sufficient to originate the process of metastasis, but that after destruction of the primary tumour, only those metastases which contain >3,000 cells continue to proliferate. In this case, removal of the primary tumour might be considered as a type of immunotherapy, as stated by Morton & Wells (1981)

The validity of these two interpretations is questionable.

(iii) Another possibility is that during the very first steps of its history, the metastasis grows much faster than when it is detected. In this hypothesis, metastases growth is characterized by a period of very rapid proliferation followed by an exponential phase. Such a situation has been observed with a transplanted murine melanoma (Steel, 1977, Figure 1.11).

Some considerations support this hypothesis. Tumours contain stem cells and non-stem cells (transient cells, end cells). Only stem cells are capable of an unlimited proliferation. In human tumours the proportion of stem cells is small. However, this proportion, estimated a long time after tumour initiation may not reflect the situation at the start. The metastasis may originate from one stem cell with a period of rapid proliferation corresponding to a filling of the non-stem cell compartment (See MacKillok *et al.*, 1983).

In human tumours, the proportion of stem cells has been estimated as about 1/1,000 by Trott *et al.* (1984). The similarity between the total number of cells per stem cell (1,000) and the estimated value of

V0M (3,000) is noteworthy. A possible relation between the apparent growth duration and the proportion of stem cells has to be investigated.

In summary, the concept of 18 DTM and exponential growth of metastases does not necessarily mean that the initial metastases volume is 3,000 cells. It is more an operational concept because the back extrapolation over the entire history of the metastasis is not valid and may overestimate the initial metastasis volume.

*Previous model*

Slack *et al.* (1969) have proposed a model to describe the natural history of breast cancer, with the aim of fitting the proportions of metastases which appeared 18 and 60 months after the treatment of the primary tumour. They assumed that tumour growth is exponential, starting from one cell, and that tumours and metastases have identical growth rates. Using their model, it was necessary to postulate the existence of two categories of breast cancers differing by their doubling times (1.4, and 0.7 months), as well as by the relative risks of metastatic dissemination (1:1.9) and nodal involvement (1:8).

Concerning the characteristics of these populations, the estimated tumour and metastases doubling times are much shorter than the measured values (Tubiana *et al.*, 1975; and Table I). Moreover, it has been found that the distribution of the doubling time is lognormal (Charbit *et al.*, 1971; Steel, 1977) and not bimodal, as it would be if there were two groups of breast cancers.

Finally, with our model, it is not necessary to postulate the existence of two populations of breast cancers. Concerning this point, the result of Slack *et al.* (1969) seems artefactual. This is probably due to the fact that Slack *et al.* (1969) did not account for variability in the parameters.

Data concerning scar recurrences can be examined on the light of our results. Philippe & Le Gal (1968) and later Pearlman (1976) estimated the doubling times of recurrences assuming an exponential growth starting from one single cell. They divided the delay between surgery and recurrence by 30 (number of DT from one cell up to 1 g). The median recurrence doubling time was estimated as 21 days by Pearlman, and 29 days by Philippe & Le Gal. The present model suggests that the delay should have been divided by 18, which leads to median recurrence doubling times of 35 and 49 days, values which are close to the estimated pulmonary metastasis doubling time.

*Consistency of our data*

The data used in this model, were obtained from various sources. In order to assess the possible effect of biased data on our results, we have performed simulations with modified distribution parameters. As shown in Table III, the adjustment conditions were only slightly modified. In all cases, it must be noted that R1 is negative, and R2 positive. The estimated metastases growth duration is always approximately the same.

Results concerning the age of the metastases are not modified.

*Relations between the parameters*

A determinist relationship has been assumed between metastasing tumours and metastases doubling times. The results are only slightly

**Table III** Adjustment conditions as a function of the parameters of the various distributions. The parameters given in the first line are those of the literature. In the subsequent lines the modified parameter is indicated by (+)

| *Parameters* | | | | *Results* | | | |
|---|---|---|---|---|---|---|---|
| *M DT (months)* | *SD DT* | *M DTM (months)* | *SD DTM* | *R1* | *R2* | *Duration DTM (months)* | *r* |
| 7 | 0.72 | 2.5 | 0.85 | −0.3 | 0.3 | 18.3 (45.9) | 2.2 |
| 5.5(+) | 0.72 | 2.5 | 0.85 | −0.4 | 0.5 | 18.3 (45.9) | 1.7 |
| 9 (+) | 0.72 | 2.5 | 0.85 | −0.2 | 0.2 | 21.6 (54.0) | 3.2 |
| 7 | 0.72 | 2.0(+) | 0.85 | −0.3 | 0.3 | 23.3 (46.6) | 2.9 |
| 7 | 0.72 | 4.0(+) | 0.85 | −0.3 | 0.3 | 11.6 (46.5) | 1.5 |
| 7 | 0.60(+) | 2.5 | 0.85 | −0.2 | 0.2 | 18.3 (45.9) | 2.5 |
| 7 | 0.85(+) | 2.5 | 0.85 | −0.4 | 0.4 | 19.1 (47.8) | 2.1 |
| 7 | 0.72 | 2.5 | 0.70(+) | −0.4 | 0.4 | 19.9 (49.8) | 2.2 |
| 7 | 0.72 | 2.5 | 1.00(+) | −0.2 | 0.2 | 18.3 (45.9) | 2.4 |

*r* is the ratio between the doubling times of metastasising tumours and of metastases; M is the median value; SD is the standard deviation of the logarithms of the values; Duration is the metastases growth duration (DTM: number of DTM; months: median duration in months).

modified if a correlation of 0.7 (instead of 1) is set between these doubling times (R1, R2, the estimated metastases growth duration and the ages of the metastases are not modified, but the fit of the big tumours metastases appearance curves is worse).

The correlation between tumour volume at diagnosis and doubling time (R1) is found equal to −0.3. With such a small value, it is not surprising that most authors, particularly Fournier *et al.* (1980) who studied 147 tumours, did not find significant correlations between volume and doubling time. However, Kusama *et al.* (1972) reported that large tumours have, on the average, a shorter doubling time than small tumours. Moreover labelling indices (LI) are slightly higher in large tumours, but the difference is small and not significant (Tubiana *et al.* 1981; Meyer *et al.*, 1983). On the other hand, the −0.3 value is the same as that deduced by Atkinson *et al.* (1983) from data on primary tumour size at diagnosis.

The positive correlation (R2) between tumour doubling time and threshold volume is consistent with observations concerning the prognostic value of the LI: rapidly proliferating tumours disseminate more often than less proliferating ones (Tubiana *et al.*, 1981, 1984; Gentilli *et al.*, 1981; Meyer *et al.* 1983).

The ratio between the median doubling times of the metastasising tumours and of the metastases is 2.2. The discrepancy between this value and the crude ratio between the median doubling times of tumours and metastases (Table I) is explained by the shorter doubling times of metastasising tumours.

### Conclusion

Previous models have not taken into account the relationship between tumour volume at treatment and metastatic dissemination probability; neither did they consider the variability of doubling times of tumours and metastases, nor the fact that the growth rates of tumours and metastases are, on the average, different.

The metastases growth duration, in our model, is not fixed *a priori*, but estimated. This duration is found equal to 18 DTM, that is about half the duration estimated with an exponential growth starting from one single cell.

The occult history of metastases (on the average 3.8 years) is found shorter than usually assumed (17 years if the metastases growth is supposed the same as the primary tumour one). A 30% reduction in metastases incidence is predicted if the primary tumours are treated 12 months earlier, which is in accordance with the results of screening programs. This concordance encourages us to explore further the possible uses of such models for other tumour sites.

We wish to express our gratitude to Drs. N. Blackett and J.Y. Mary for their careful reading of the text and their helpful comments and suggestions.

### Appendix

*Correlation between V1T and V0T*

The partial correlation between V1T and V0T is considered equal to zero when DT is fixed (notation: R V1T V0T/DT = 0).

This restriction can be written:

$$\mathrm{R\,V1T\,V0T/DT} = 0 = \frac{K - \mathrm{R1\,R2}}{\sqrt{(1-\mathrm{R1}^2)(1-\mathrm{R2}^2)}}, \quad (1)$$

where $K$ is the correlation between V1T and V0T, R1 the correlation between V1T and DT and R2 the correlation between V0T and DT.

This restriction implies that

$$K = \mathrm{R1\,R2}.$$

The value of $K$ is independent of the parameters of the threshold volume distribution.

*Distribution of the threshold volume*

In a previous paper (Koscielny *et al.*, 1984), independence was assumed between the volume at diagnosis V1T and V0T. Under this assumption, the threshold volume (V0T) distribution was estimated from the relationship between the cumulated proportion ($p$) of metastases at long term and the logarithm of the tumour volume (V1T) at treatment. This relation, interpreted in terms of quantal response (Finney *et al.*, 1964) can be written:

$$Y = \mathrm{probit}(p) - 5 = \frac{\mathrm{V1T} - (\mu e\mathrm{V0T})}{(\sigma e\mathrm{V0T})}, \quad (2)$$

$$Y = a\,\mathrm{V1} + b; \quad (3)$$

the coefficients $a$ and $b$ are respectively equal to $1/\sigma e\mathrm{V0T}$ and $-\mu e\mathrm{V0T}/\sigma e\mathrm{V0T}$ where $\mu e\mathrm{V0T}$ and $\sigma e\mathrm{V0T}$ are the mean and the SD of the distribution of the logarithm of the threshold volume estimated from the data.

When $K$ (correlation between V1T and V0T) is not nil, the distribution of V0T must be recalculated conditionally to the value of $K$.

$\mu\mathrm{V0T}$ and $\sigma\mathrm{V0T}$ are the mean and the SD of V0T distribution, conditionally to the value of $K$.

$$\mu\mathrm{V0T/V1T} = \mu\mathrm{V0T} + K\sigma\mathrm{V0T}/\sigma\mathrm{V1T}(\mathrm{V1T} - \mu\mathrm{V1T}), \quad (4)$$

$$\sigma\mathrm{V0T/V1T} = \sigma\mathrm{V0T}\sqrt{1-K^2}. \quad (5)$$

A relationship similar to (2) is

$$Y = \frac{\text{V1T} - \mu\text{V0T/V1T}}{\sigma\text{V0T/V1T}}. \quad (6)$$

The parameters $a$ and $b$ now refer to:

$$a = \frac{1-(K\sigma\text{V0T}/\sigma\text{V1T})}{\text{V0T}\sqrt{1-K^2}}. \quad (7)$$

$$b = -\frac{\mu\text{V0T} - K\sigma\text{V0T}/\sigma\text{V1T}\,\mu\text{V1T}}{\text{V0T}\sqrt{1-K^2}}, \quad (8)$$

where

$$\sigma\text{V0T} = \frac{\sigma e\text{V0T}\sigma\text{V1T}}{\sigma\text{V1T}\sqrt{1-K^2} + K\sigma e\text{V0T}}, \quad (9)$$

$$\mu\text{V0T} = \frac{\mu e\text{V0T}\sigma\text{V1T}\sqrt{1-K^2} + K\sigma e\text{V0T}\,\mu\text{V1T}}{\sigma\text{V1T}\sqrt{1-K^2} + K\sigma e\text{V0T}}. \quad (10)$$

*Quantification of the growth*

With the Gompertzian growth function, the relation between volume and time (Gatton *et al.*, 1978) is:

$$\text{Ln}(\text{V}t) = a - b\exp(-ct), \quad (11)$$

where

$$a = \text{Ln(Vm)}; \quad (12)$$

Vm represents the maximum asymptotic volume (volume that would be reached after an infinite period of growth), and

$$b = \text{Ln(Vm/Vi)}. \quad (13)$$

Vi is the initial volume (at time 0).

The doubling time at time $t$ is equal to:

$$\text{DT}t = \text{Ln}(2)/bc\exp(-ct). \quad (14)$$

From (11) and (12),

$$b\exp(-ct) = \text{Ln}(\text{Vm/V}t). \quad (15)$$

From (13) and (15),

$$ct = \text{Ln}(\text{Ln(Vm/Vi)}/\text{Ln}(\text{Vm/V}t)). \quad (16)$$

From (14) and (15),

$$c\,\text{DT}t = \text{Ln}(2)/\text{Ln}(\text{Vm/V}t). \quad (17)$$

The ratio (16)/(17) gives the value of $t$ as a function of DT$t$:

$$t = \text{DT}t\,\text{Ln}\{\text{Ln(Vm/Vi)}/\text{Ln}(\text{Vm/V}t)\} \quad \text{Ln}(\text{Vm/V}t)/\text{Ln}(2). \quad (18)$$

Concerning doubling times, we can write

$$c\text{DT}^\circ = \text{Ln}(2)/\text{Ln}(\text{Vm/V}^\circ). \quad (19)$$

where DT° is the doubling time when the volume is V°.

From (17) and (19),

$$\text{DT}^\circ = \text{DT}t\,\text{Ln}(\text{Vm/V}t)/\text{Ln}(\text{Vm/V}^\circ). \quad (20)$$

**References**

ALEXANDER, P. (1985). Do cancers arise from a single transformed cell or is monoclonality a late event in carcinogenesis? *Br. J. Cancer,* **51,** 453.

ATKINSON, E.N., BARTOSZYNSKI, R., BROWN, B.W. & THOMPSON, J.R. (1983). On estimating the growth function of tumours. *Math. Biosc.,* **67,** 145.

BREUR, K. (1976). Conference on the *Biological Behaviour of Tumour and Chronobiology in Tumours,* Oslo (unpublished, quoted in Tubiana, 1982).

CHARBIT, A., MALAISE, E.P. & TUBIANA, M. (1971). Relationship between the pathological nature and the growth rate of human breast cancer. *Eur. J. Cancer,* **7,** 307.

COMBES, P.F., DOUCHEZ, J., CARTON, M. & NAJA, A. (1968). Etude de la croissance des métastases pulmonaires humaines comme argument objectif d'évaluation du pronostic et des effets thérapeutiques. *J. Radiol. Electrol.,* **49,** 893.

EDWARDS, M.H., BAUM, M. & MAGARAY, C.J. (1972). Regression of axillary lymph nodes in cancer of the breast. *Br. J. Surg.* **69,** 776.

FINNEY, D.J. (1964). Statistical method in biological assay (2nd edition). Charles Griffin & Co.: London.

FOURNIER, D.V., WEBER, E., HOEFFKEN, W., BAUER, M., KUBLI, F. & BARTH, V. (1980). Growth rate of 147 mammary carcinomas. *Cancer,* **45,** 2198.

GATTON, R.J., APPLETON, D.R. & ALWISWASY, M.K. (1978). The measurement of tumour growth rates. In *Biomathematics and Cell Kinetics,* Valleron, A.-J. & Macdonald, P.D.M. (eds) p. 325. Elsevier/North-Holland: Amsterdam.

GENTILLI, C., SANFILIPPO, O. & SILVESTRINI, R. (1981). Cell proliferation and its relationship to clinical features and relapse in breast cancers. *Cancer,* **48,** 974.

GHERSHON-COHEN, J., BERGER, S.M. & KLICKSTEIN H.S. (1963). Roentgenography of breast cancer moderating the concept of 'Biologic predeterminism'. *Cancer,* **16,** 961.

HAMMERSLEY, J.M. & HANDSCOMB, D.C. (1967). Monte Carlo Methods. Methuens' monographs: London.

HEUSER, L., SPRATT, J.S. & POLK, H.C. (1979). Growth rates of primary breast cancers. *Cancer,* **43,** 857.

IGOT, J.P. & LE GAL, Y. (1968). Age des adénopathies métastatiques dans le cancer mammaire. *Ann. Anat. Path.* **13,** 449.

KOSCIELNY, S., TUBIANA, M., LÊ M.G. & 4 others (1984). Relationship between the size of the primary tumour and the probability of metastatic dissemination. *Br. J. Cancer.*, **49**, 709.

KUSAMA, S., SPRATT, J.S., DONEGAN, W.L., WATSON, F.R. & CUNNINGHAM, C. (1972). The gross rates of growth of human mammary carcinoma. *Cancer*, **30**, 594.

MACKILLOP, W.J., CIAMPI, A., TILL, J.E. & BUICK, R.N. (1983). A stem cell model of human tumor growth: Implications for tumor cell clonogenic assays. *J. Natl Cancer Inst.*, **70**, 9.

LÜNDGREN B. (1977). Observations on growth rate of breast carcinomas and its possible implications for lead time. *Cancer*, **40**, 1722.

MALAISE, E.P., CHAVAUDRA, N., CHARBIT, A. & TUBIANA, M. (1972). The relationship between growth rate, labelling index and histological type of tumours. *Eur. J. Cancer*, **10**, 451.

MEYER, J.S., FRIEDMAN, E., McCRATE, M. & BAUER, W.C. (1983). Prediction of early course of breast carcinoma by thymidine labelling. *Cancer*, **51**, 1879.

MORTON, D.L. & WELLS, S.A. (1981). Immunology of neoplastic disease. In *Davis-Christofer Textbook of Surgery*, Sabiston, D.C. (ed). Saunders: Philadelphia.

PEARLMAN, A.W. (1976). Breast cancer – Influence of growth rate on prognosis and treatment evaluation. *Cancer*, **38**, 1826.

PHILIPPE, E. & LE GAL Y. (1968). Growth of seventy-eight recurrent mammary cancers. *Cancer*, **21**, 461.

SCHWARTZ, M. (1961). A biomathematical approach to clinical tumor growth. *Cancer*, **14**, 1272.

SHAPIRO, S., VENET, W., STRAX, P., VENET, L. & ROESER, R. (1982). Ten- to fourteen-year effect of screening on breast cancer mortality. *J. Natl. Cancer Inst.* **69**, 349.

SLACK, N. H., BLUMENSON, L.E. & BROSS, I.D.J. (1969). Therapeutic implications of a mathematical model characterizing the course of breast cancer. *Cancer*, **24**, 960.

SLEMMER, G.T. (1979). Host response to antigenic tissues during progression and metastasis of breast neoplasia. In *Tumor Progression*, Crispen, R.G. (ed) p. 3. Elsevier/North-Holland: New York.

SPRATT, J.S. & SPRATT, T.L. (1964). Rates of growth of pulmonary metastases and host survival. *Ann. Surg.* **159**, 161.

STEEL, G.G. (1977). Growth kinetics of tumours. University Press: Oxford.

TROTT, K.R., MACIEJEWSKI, B., PREUSS-BAYER, G. & SKOLYSZEWSKI, J. (1984). Dose-response and split-dose recovery in human skin cancer. *Radiotherapy and Oncology*, **2**, 123.

TUBIANA, M., CHAUVEL, P., RENAUD, A. & MALAISE, E.P. (1975). Vitesse de croissance et histoire naturelle du cancer du sein. *Bull. Cancer*, **62**, 341.

TUBIANA, M., PEJOVIC, M.J., RENAUD, A., CONTESSO, G., CHAVAUDRA, N., GIOANNI, J. & MALAISE, E.P. (1981). Kinetic parameters and the course of the disease in breast cancer. *Cancer*, **47**, 937.

TUBIANA, M. (1982). Cell kinetics and radiation oncology. *Int. J. Radiat. Oncol. Biol. Phys.*, **8**, 1471.

TUBIANA M., PEJOVIC, M.H., CHAVAUDRA, N., CONTESSO, G. & MALAISE, E.P. (1984). The long-term prognostic significance of the Thymidine Labelling Index in breast cancer. *Int. J. Cancer*, **33**, 441.

WALTER, S.D. & DAY, N.E. (1983). Estimation of the duration of a pre-clinical disease state using screening data. *Am. J. Epidemiol.* **118**, 865.